# Thermomechanical Behaviour and Interface of Overmoulded Soft Thermoplastic Vulcanizate Elastomers

**DOI:** 10.3390/ma14195704

**Published:** 2021-09-30

**Authors:** Pierre Le Mouellic, Sylvain Charlès, Jean-Benoît Le Cam, Nicolas Boyard, Jean-Luc Bailleul, Thierry Gaudry, Jean-Marc Veillé

**Affiliations:** 1 Cooper Standard, Route des Eaux, 35503 Vitré, France; Thierry.GAUDRY@cooperstandard.com (T.G.); Jean-Marc.VEILLE@cooperstandard.com (J.-M.V.); 2 Laboratoire de Thermocinétique de L’école Polytechnique de L’université de Nantes, UMR CNRS 6607, rue Christian Pauc, BP 50609, CEDEX 3, 44306 Nantes, France; nicolas.boyard@univ-nantes.fr (N.B.); Jean-Luc.Bailleul@univ-nantes.fr (J.-L.B.); 3 Université de Rennes 1, Institut de Physique UMR 6251 CNRS de Rennes 1, Campus de Beaulieu, Bât. 10B, CEDEX, 35042 Rennes, France; sylvain.charles@univ-rennes1.fr (S.C.); jean-benoit.lecam@univ-rennes1.fr (J.-B.L.C.)

**Keywords:** overmoulding, vulcanizate thermoplastic elastomer, polymer interface, polymer junction

## Abstract

The influence of melt injection temperature on the thermomechanical behaviour of soft–soft overmoulded vulcanized thermoplastic elastomers (TPV) with different elastic properties was studied. Samples with two different overmoulding temperatures were tested under uniaxial loading conditions. The full deformation and temperature fields in each TPV were determined using digital image correlation technique and infrared thermography, respectively. The maximum interface strength was found to be equal to 70N for a maximum injection temperature of $260{\;}^{\circ }C$, which is consistent with the fact that high temperatures promote interdiffusion between the molten TPV and the TPV insert. The two TPV have different stiffness, leading to a significant change of the interface position along the specimens during stretching and to a significant necking in the softer material. The zone of influence of the interface in terms of stretch gradient is very different in size from one TPV to the other. In addition, thermal investigations have shown that the elasticity of the two TPV is due to both entropic and non-entropic effects, the former being the most significant at large strains.

## 1. Introduction

Injection moulding is a suitable technique for the production of numerous, high-quality and complex geometry parts in short times. Overmoulding is an injection technique that consists of joining two polymeric parts without any adhesives. This injection technique is now used in several sectors such as automotive, medical or packaging. For the automotive market, more especially in the weather seals, overmoulding is used to joined extrudate profiles. Soft–soft combinations are often used to answer the specific needs of the customer such as insulation from noise, water and dust. The soft–soft combinations were classically made of ethylene-propylene-diene-monomer (EPDM) rubber, but recent trends show increasing use of thermoplastic elastomer vulcanizate (TPV). This type of thermoplastic elastomer (TPE) is produced by dynamic vulcanization [1], which leads to the elastomer crosslinking during the blending with a thermoplastic polymer [2]. The resulting morphology is an elastomeric dispersed phase into a thermoplastic matrix. The elastomeric phase is usually composed of EPDM, and the thermoplastic phase is polypropylene (PP) [3]. These materials have a great interest since they exhibit the features of elastomers with the processability of thermoplastics, allowing short cycle times and therefore a productivity increase. During the removal of the overmoulded part from the mould, the assembly on the vehicle or during the use, debonding at the interfaces can occur. It is then important to understand the adhesion mechanisms at TPV interfaces and to analyze the mechanical behaviour of these junctions.

Overmoulding fusion bonding between semicrystalline thermoplastics and TPE has been the subject of attention in various studies. Candal et al. [4] studied the bonding strength of TPV on a polypropylene substrate by the Essential Work of Interfacial Fracture (EWIF) [5]. They varied the overmoulding conditions and showed that increasing the melt and mould temperatures lead to higher adhesion since these parameters improve the wettability and interdiffusion of the TPV on the PP substrate. The authors used a 190 °C injection temperature and with the EWIF method the interfacial strength was found to be 2 kJ.m${}^{-2}$, whereas, for an injection temperature of $260{\;}^{\circ }C$ they found an interface strength of 6 kJ.m${}^{-2}$. They also showed that an increase in holding pressure decreased adhesion. The bonding between thermoplastic elastomer and Polyamide 12 with 0 to 50% glass fibre contents by a peeling test has been investigated by Persson et al. [6]. The authors showed that adhesion increased with increasing melt temperature and injection rate for a certain range. Rosa-Sierra et al. [7] have studied the adhesion between methylmethacrylate-butadiene-styrene copolymer (MABS) and thermoplastic polyurethane (TPU) by peeling and tensile tests. The roughness of the insert was shown to be the most influential parameter on the adhesion strength. The authors used an insert roughness of 31.33μm and with the peel test the interfacial strength was found to be 31.9 kJ.m${}^{-2}$, whereas with a roughness of 3.24μm the adhesion strength was found to be 5.2 kJ.m${}^{-2}$. The authors also showed the increase of adhesion with the overmoulding temperature. Other studies [8,9] focus on the influence of melt injection temperature and holding pressure on the adhesion strength between ethylene or octane copolymers on PP homopolymer substrate by peeling tests. It is demonstrated that the adhesion strongly depends on the interface temperature and thus on the development of entanglements between the two molten polymers. Weng et al. [10] have summarized the fundamental reasons for adhesion of an overmoulded TPE. They explain that the overmoulding process can be divided into three stages: wetting, diffusion of polymer chains and crystallization.

Wetting refers to the notion of intimate contact. The melted TPE is injected into a mould in which the TPE insert to be overmoulded has been previously positioned. The contact between both TPEs is not perfect due to the surface roughness of the insert. The notion of intimate contact is used to describe the evolution of physical contact between the two TPEs. The degree of intimate contact increases when the asperities of the TPE insert squeeze under the application of pressure and polymer flow of the injected TPE. Models to describe the evolution of intimate contact have been developed [11,12,13], and generally show that the time required to achieve full intimate contact $t_{ic}$ is proportional to the viscosity $\eta_{0}$ and inversely proportional to the applied pressure *P* [12]:

(1) $$t_{ic}\propto \frac{\eta_{0}}{P}$$

The intimate contact time refers to the time from the beginning of the process until the time for which polymer chains diffuse and create the junction strength. In other words, the intimate contact time precedes the healing time. The injection process involves melted polymer with low viscosities and high pressure. It is, therefore, possible to neglect the time necessary to achieve intimate contact (Equation (1)). This process is followed by healing which refers to the diffusion of polymer (PP) chains across the interface. These two processes are coupled because healing can occur only across the zones where intimate contact is established. The process of healing is described from the theory of De Gennes [14] taken up by Doi and Edwards [15] and Wool [16]. The theory of De Gennes describes the movement of macromolecular chains in an entangled system constrained by the presence of neighbouring chains. A chain is then considered to be confined in a permanent and undeformable tube. The behaviour of the chain is anisotropic: lateral movements are not possible, and the polymer relaxes by monodirectional movements along its tube. The relaxation process of the polymer chain is associated with a characteristic time called reptation time depending on the temperature and molar mass. This characteristic time represents the time necessary for the chain to exit its initial tube and forget its initial configuration. Bastien and Gillespie [17] introduce the degree of healing (Equation (2)) for non-isothermal temperature history at melted polymer interface as follows:

(2) $$D_{h}=\frac{\sigma }{\sigma_{\infty }}=\sum_{i=0}^{t_{p}/\Delta t}\frac{t_{i+1}^{1/4}-t_{i}^{1/4}}{t_{rep}^{*\;1/4}}$$

where σ is the Cauchy fracture stress, $\sigma_{\infty }$ is the fracture stress of a fully healed interface, $t_{p}/\Delta t$ represents the division of the thermal history in time intervals characterized by an average temperature $T^{*}$ for which the reptation time $t_{rep}^{*}$ is determined. The final performance of the overmoulded part is the combination of the contribution of wetting, diffusion and crystallization. If hard-soft combinations are studied in the literature, soft–soft combinations seem to be few to the author’s knowledge.

The difference in elastic properties of the overmoulded TPV made their global mechanical behaviour complex. Indeed, contrarily to a soft-hard configuration, soft–soft configuration leading to large and very different deformation levels in the two materials, this induces a significant change in the position of the interface and in the loading applied locally in this zone. Even though mechanical models and experimental tests are reported in the literature for improving the prediction and analysis of the behaviour of materials at interfaces, such soft–soft configuration has never been considered to our best knowledge, which makes the innovative character of the present study. As a summary, this paper reports the first study on mechanical properties and interface of two TPV with very different stiffnesses. To do so, an original experiment is developed to characterize the material deformation processes at both the global and the local scales. The results reported in this study bring therefore new information on how characterizing the interface between two different TPV materials and data to better model interface effects in a continuum media. Therefore, the present work aims to study the thermomechanical behaviour of soft–soft combinations made of overmoulded TPV. In the present study, two TPV overmoulded at two different temperatures are considered. Two types of tests were performed at room temperature: A monotonous tensile test until failure and cyclic load–unload tests with a constant amplitude. The experimental setup and the theoretical framework for thermal and kinematic field measurements are detailed in Section 2. Section 3 gives the results obtained in terms of kinematic and thermal field. The mechanical strength of the assembly is discussed regarding the overmoulding temperature.

## 2. Materials and Methods

### 2.1. Materials and Specimens Elaboration

#### 2.1.1. Materials

The materials considered here are two polypropylene-ethylene-propylene-diene-monomer (PP-EPDM)-based thermoplastic elastomers (TPV) called TPV1 and TPV2. TPV1 is an injection moulding grade, TPV2 is an extrusion grade. Both TPVs are composed of several components, each of them providing different properties to the material:

1. an elastomer (EPDM), for flexibility, deformation at break and impact resistance,
2. a thermoplastic (PP), for the processing and the stiffness,
3. fillers, for tunning specific mechanical and rheological properties [18]. Generally, these fillers are carbon black [19] or silica [20],
4. a vulcanization agent, here phenolic resin,
5. antioxidant agents.

It should be noted that each TPV of the present study is obtained by a dynamic vulcanization process such as described in [21], which consists of mixing the two melt phases (PP and EPDM) and then in vulcanizing the EPDM. In this process, a phase inversion is obtained in the sense that the PP phase is initially dispersed in the EPDM phase and after the vulcanization, EPDM is dispersed in the PP phase. The final morphology of the mixing is determined by the relative amount of the two phases, their viscosity, the crosslink density and the type and amount of the vulcanization [22,23]. The main properties of TPV1 and TPV2 are given in Table 1.

The morphology of the two TPV has been investigated thanks to a Bruker Multimode 8 NanoscopeV Atomic Force Microscope (AFM) in tapping mode. The scan sizes were 20μm and 40μm. The scan rate was 0.977Hz. The cantilever parameters were a spring constant of 0.3N/m and a tip radius of 10nm. The overmoulded samples were previously cryo-microtomed at $-80{\;}^{\circ }C$. The images were processed using Gwyddion software.

The specific heat ($C_{p}$) of both TPV has been determined by differential scanning calorimetry (Q200, TA Instruments). The measurements were made at a heating rate of 10K/min between $50{\;}^{\circ }C$ and $200{\;}^{\circ }C$. The specific volume $V_{sp}$, in m${}^{3}$/kg, was measured with a home-made PVT device [24] for 10MPa and 50MPa at a cooling rate of $2{\;}^{\circ }C/\min$. Thermal conductivity *k* in W/(m.K) was measured for the temperature ranging between $23{\;}^{\circ }C$ and $260{\;}^{\circ }C$ thanks to a home-made guarded hot plate [25].

The main characteristics of the PP constitutive of the TPVs matrices are listed in Table 2. Polypropylene 1,2 (PP1,2) is an ethylene-propylene copolymer and is representative of the matrix of TPV1 and PP1 is a homopolymer PP and is representative of the TPV2 matrix. These analyses were performed on a “Malvern viscotek” steric exclusion chromatography. The solvent was the 1,2,4-Trichlorobenzene. The elution speed of the solvent was fixed at 1mL/min.

The linear viscoelastic properties of the PP representative of TPV1 and TPV2 matrices were determined using a strain-controlled rheometer HAAKE MARS III (Thermo Scientific, Waltham, MA, USA) equipped with a 25mm parallel plate geometry and a 1mm gap. Dynamic frequency sweep measurements were performed for frequencies ranging between 0.01rad/s and 100rad/s, γ = 5% for different temperatures between $200{\;}^{\circ }C$ and $230{\;}^{\circ }C$.

#### 2.1.2. Overmoulding Protocol

**Figure 1:**
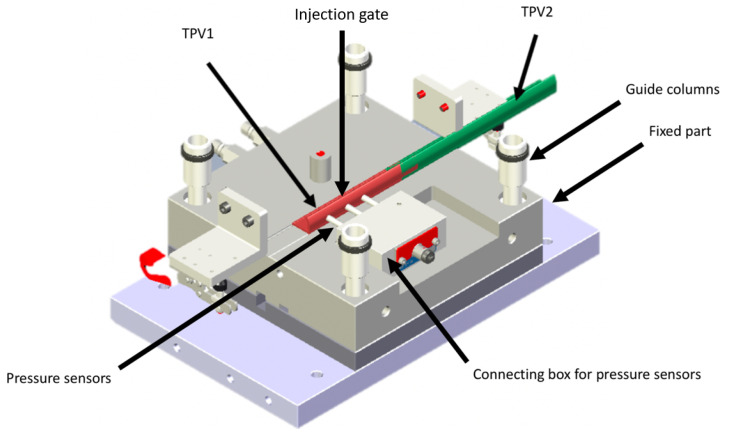
Overmoulding mould. The green and red profiles represent TPV2 and TPV1, respectively. Two pressure sensors are located at the inlet and at the outlet of the cavity. TPV1 is injected through a 3mm diameter sprue.

TPV2 were extruded with a FAIREX extruder (barrel diameter 60 mm). The extruded profiles were cut with a razor blade and stored at $23{\;}^{\circ }C$ for 24 h and 50% relative humidity. Their dimensions were 60mm long, 20mm wide and 2mm thick. TPV1 were dried at $85{\;}^{\circ }C$ in a Moretto drier for 2 h. These profiles were then inserted in a specific mould, which is presented in Figure 1, and were overmoulded (LWB STEINL, 300 kN injection unit) with TPV1. The injection temperatures for TPV1 were set at $190{\;}^{\circ }C$ or 260with an injection rate of 12 cm${}^{3}$/s, a hydraulic holding pressure of 0.7MPa, a holding pressure time of 5 s and a mould temperature of $40{\;}^{\circ }C$. The moulding cavity is instrumented with two pressure sensors Kistler© (type 6182CAG) positioned at 10mm and 50mm from the gate, respectively.

#### 2.1.3. Specimen Preparation

**Figure 2:**
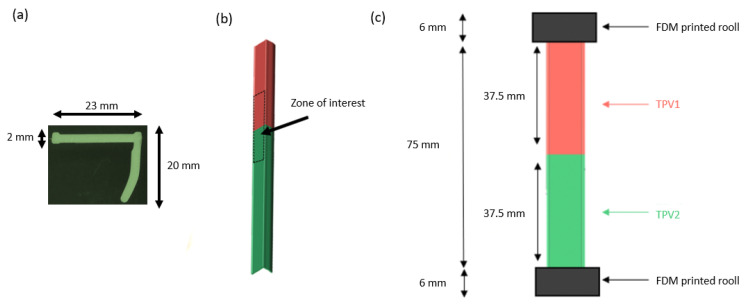
(**a**) Cross-section of the profile. (**b**) Zone of interest centred on the interface. (**c**) Dimensions of the specimens. FDM printed rolls of 6mm diameter were glued to the ends of the specimens.

The cross-section of the parts is shown in Figure 2a. Once overmoulding was performed, rectangular bi-material specimens were cut from the overmoulded parts with a razor blade in the zone of interest centred on the interface (see Figure 2b). As shown in Figure 2c, their dimensions were 75 mm long, 15 mm wide and 2 mm thick. Fused Deposition Modelling (FDM) printed rolls of 6 mm in diameter were glued (Loctite 406, Henkel, Düsseldorf, Germany) to the ends of the specimen. Such rolls prevent the specimens from slipping into the jaws, which shape has been designed accordingly [26,27].

### 2.2. Mechanical Characterization

**Figure 3:**
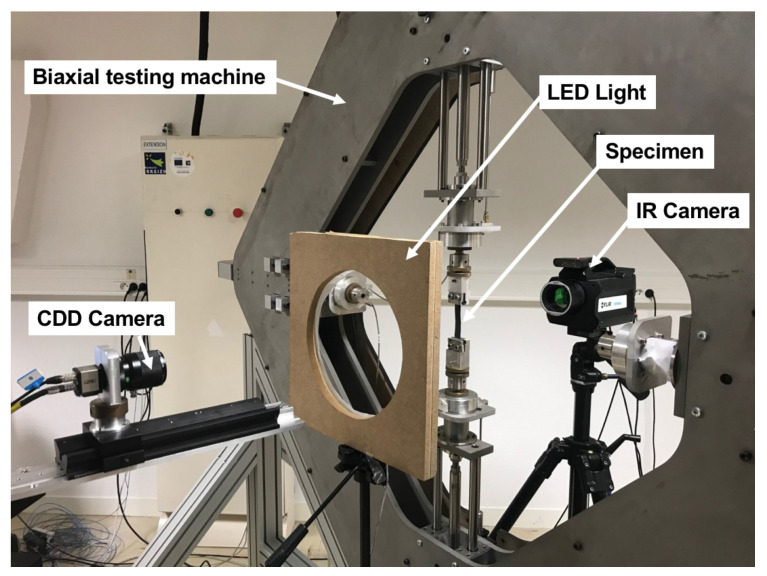
Photography of the experimental setup for mechanical characterization.

Figure 3 gives an overview of the experimental setup. It is composed of optical and infrared cameras, one on each side of a home-made biaxial testing machine. The tensile machine used is composed of four independent RCP4-RA6C-I-56P-4-300-P3-M (IAI) electrical actuators. They were driven by a PCON-CA-56P-I-PLP-2-0 controller and four PCON-CA (IAI) position controllers. The actuators were piloted by an in-house LabVIEW program. Two load cells measure the force in the perpendicular direction. Only one loading direction of the biaxial tensile machine was used for the present study and the specimens were mounted in the vertical direction. Two types of loading were applied. The first one corresponds to a monotonous tensile test until specimen failure at a rate of 36mm/min per actuator. The second one corresponds to one cyclic loading at the same loading rate and at a total displacement of 8mm and 25mm for specimens overmoulded at $190{\;}^{\circ }C$ and $260{\;}^{\circ }C$, respectively. The displacements have been selected according to the maximum displacement reached during tensile test at failure.

### 2.3. Full Kinematic Field Measurement

Images of the specimen surface were recorded at a frame rate equal to 10 Hz with an IDS camera equipped with a 55 mm telecentric objective. The charge-coupled device (CCD) of the camera has 1920 × 1200 joined pixels. The displacement field at the specimen surface was determined using the digital image correlation (DIC) technique. The software used for the correlation process was SeptD [28]. Hardware and analysis parameters are summarized in Table 3 and Table 4, respectively.

### 2.4. Deformation Gradient Tensor Computation

As explained in [27], the $F_{ij}$ components of the deformation gradient tensor are determined at the centre of the square elements, which are composed of four Zones Of Interest (ZOI). Each corner of the square element corresponds to a ZOI centre defined in the undeformed DIC grid. Within each square element *i*, the horizontal and vertical displacements, ${}^{i}U$ and ${}^{i}V$ respectively, are assumed to be bilinear functions of the coordinates (X,Y):

(3) $$\left\{\begin{array}{c} {}^{i}U\left(X,Y\right)=a+bX+cY+dXY\\ {}^{i}V\left(X,Y\right)=e+fX+gY+hXY \end{array}\right.$$

where *a*, *b*, *c*, *d*, *e*, *f*, *g* and *h* are constants obtained from the ZOI values. The displacement is interpolated at the centre of each square element, the calculation is carried out by considering that the element centre is the origin of the coordinate system. Finally, the deformation gradient tensor **F** components are calculated as follows:

(4) $$F=\left(\begin{array}{cc} \frac{\partial x}{\partial X} & \frac{\partial x}{\partial Y}\\ \frac{\partial y}{\partial X} & \frac{\partial y}{\partial Y} \end{array}\right)=\left(\begin{array}{cc} \frac{\partial X{+}^{i}U}{\partial X} & \frac{\partial X{+}^{i}U}{\partial Y}\\ \frac{\partial Y{+}^{i}V}{\partial X} & \frac{\partial Y{+}^{i}V}{\partial Y} \end{array}\right)=\left(\begin{array}{cc} 1+b & c\\ f & 1+g \end{array}\right)$$

The three principal stretches ($\lambda_{1}>\lambda_{2}>\lambda_{3}$) are defined as the square roots of the eigenvalues of the left Cauchy-Green tensor B (B = ${FF}^{t}$). $\lambda_{3}$ is deduced by assuming the material being is incompressible, i.e., $J=\det F=\lambda_{1}\lambda_{2}\lambda_{3}=1$.

### 2.5. Full Temperature Field Measurements

Measuring the thermal response associated with the mechanical response of materials provides very complementary information on the origin of the elasticity, i.e., coupling between temperature and strain, as well as on dissipative effects, typically when self-heating occurs. Two types of thermoelastic coupling have been reported in the literature for elastomeric materials: isentropic [29] and entropic coupling [30,31]. They take place concomitantly and a competition is observed between both. Thus, the thermal response of vulcanized rubber is characterized by a slight cooling during stretching in the very low stretch range (typically for stretches lower than 1.1). Such thermo-sensitivity is explained by preponderant effects of internal energy changes at low stretches [32,33,34,35,36,37,38]. At higher stretches, the competition is in favour of the entropy changes, leading to a thermoelastic inversion and a strong heating [31,39,40].

Full surfaces temperature field measurements were performed using a X6540SC FLIR IR camera, which detectors operating in wavelengths between 1.5 and 5.1μm, equipped with a focal plane array of 640 × 512 px. The integration time was equal to 2700μs and the acquisition frequency was equal to 10 Hz. The calibration of camera detectors was performed with a black body using a one-point Non-Uniformity Correction (NUC) procedure at this acquisition frequency. The thermal resolution or noise equivalent temperature difference (NETD) is equal to 20 mK for a temperature range between 5 and $40{\;}^{\circ }C$ and the spatial resolution of the thermal field was equal to 300μm/px. The IR camera is switched on several hours before testing to ensure its internal temperature to be stabilised. The emissivity of the material was set at 0.94. The emissivity has been identified from the calibration procedure detailed in [27]. The infrared camera was trigged at the same acquisition frequency as the optical camera one, i.e., 10 Hz. Due to large deformations undergone by the material, the material points observed by the IR camera move from pixel to pixel in the IR images and their motion has been compensated [40,41]. This requires first describing the kinematic and the thermal fields in the same coordinate system. In the present paper, coupled full thermal and kinematic fields measurements have been carried out on both sides of the specimen. A calibration test pattern was placed in one of the grips. The reader can refer to [27] for further information about the calibration procedure. This approach has been successfully applied for rubber [42,43,44,45,46,47,48], for Poly(methyl methacrylate) [49] and for metallic materials [50,51,52,53,54,55]. The analysis of the heat source release induced by the deformation remains delicate. Indeed, this analysis is based on a measurement of the effects (temperature variations) to go back to the causes (heat sources).

## 3. Results

### 3.1. Mechanical Analysis

#### 3.1.1. Monotonic Tensile Test until Failure

**Figure 4:**
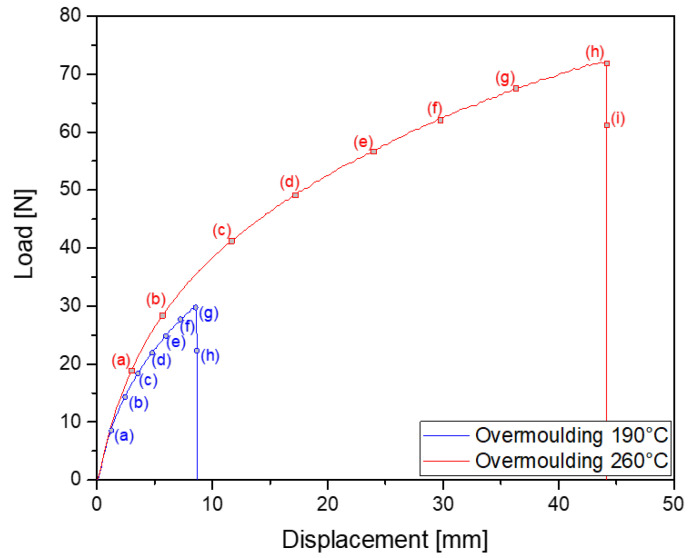
Tensile mechanical response up to failure of $190{\;}^{\circ }C$ and $260{\;}^{\circ }C$ overmoulded specimens. Letters (a) to (i) and (a) to (h) refer to the letters of images taken at different displacements and given in Figure 5 and Figure 6, respectively.

**Figure 5:**
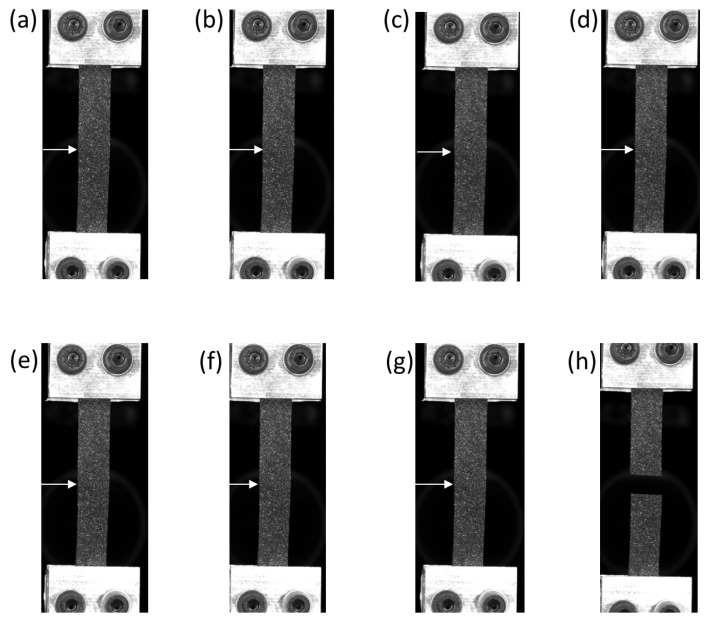
Pictures related to Figure 4, specimens overmoulded at $190{\;}^{\circ }C$. TPV1 is at the bottom and TPV2 at the top.

**Figure 6:**
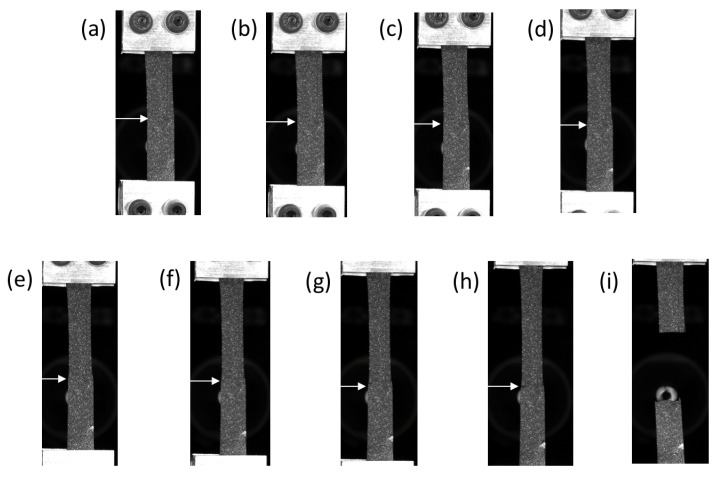
Pictures related to Figure 4, specimens overmoulded at $260{\;}^{\circ }C$. TPV1 is at the bottom and TPV2 at the top.

Figure 4 presents the mechanical behaviour during a monotonic tensile test until failure of the specimens overmoulded at $190{\;}^{\circ }C$ and $260{\;}^{\circ }C$. Figure 5 and Figure 6 present the images of the specimens overmoulded at $190{\;}^{\circ }C$ and $260{\;}^{\circ }C$ at different times of the test, respectively. These times correspond to letters a to h and a to j for $190{\;}^{\circ }C$ and $260{\;}^{\circ }C$, respectively. The interface is highlighted with a white arrow. At the beginning of the test, the shape of the curves is close. The force-displacement relationship is almost linear. For higher displacement levels, the specimens overmoulded at $260{\;}^{\circ }C$ exhibits a higher stiffness. Moreover, the displacement at break is much lower for the specimens overmoulded at $190{\;}^{\circ }C$ (8mm against 45mm for the specimens overmoulded at $260{\;}^{\circ }C$). For both overmoulding temperatures, specimen failure occurred at the interface between the two TPV. The failure occurs at a lower force in the case of specimens overmoulded at $190{\;}^{\circ }C$ than for specimens overmoulded at $260{\;}^{\circ }C$. For specimens overmoulded at $260{\;}^{\circ }C$, necking at the interface during the test is observed (Figure 6h). This may influence the strain state at the interface. Nevertheless, the spatial resolution of the full kinematic measurements did not enable us to further investigate this effect. This phenomenon is not observed for those overmoulded at $190{\;}^{\circ }C$. High relative deformations are reached for TPV2, while TPV1 shows low deformations. These remarks will be discussed in more detail in the next section.

#### 3.1.2. Mechanical Cycle

**Figure 7:**
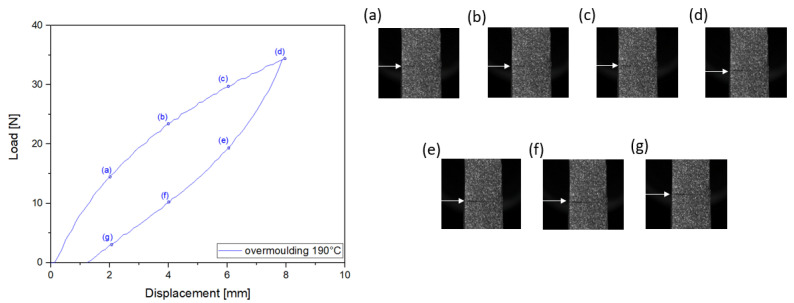
Cyclic loading 8 mm of specimens overmoulded at $190{\;}^{\circ }C$. Images (**a**–**g**) show a close-up view of the interface at different displacements during the test. TPV1 is at the bottom and TPV2 at the top.

**Figure 8:**
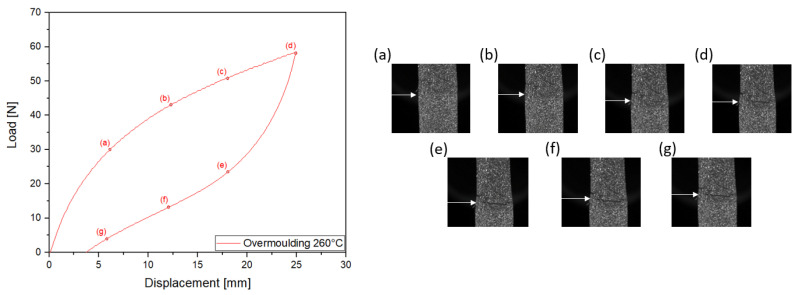
Cyclic loading 25 mm of specimens overmoulded at $260{\;}^{\circ }C$. Images (**a**–**g**) show a close-up view of the interface at different displacements during the test. TPV1 is at the bottom and TPV2 at the top. Necking can be seen for TPV1 in image (**e**).

Figure 7 and Figure 8 show the mechanical response in terms of force versus displacement during the mechanical cycle at a total displacement of 8mm for the $190{\;}^{\circ }C$ overmoulded specimens and 25mm for the $260{\;}^{\circ }C$ overmoulded specimens. For specimens overmoulded at $260{\;}^{\circ }C$, necking can be seen for TPV1 (Figure 8e), but not for TPV2. This necking cannot be seen for specimens overmoulded at $190{\;}^{\circ }C$. This has already been observed in the case of monotonous displacement test until failure (Figure 6h). This aspect will be more precisely detailed in the next section. The load levels obtained for the mechanical cycle are similar to the one obtained previously (Figure 4). The mechanical response exhibits a hysteresis for both specimens. This hysteresis loop can be associated with viscosity and/or damage. In the case of $190{\;}^{\circ }C$ overmoulded specimens, the residual strain is 1.67% against 5% for the $260{\;}^{\circ }C$.

**Figure 9:**
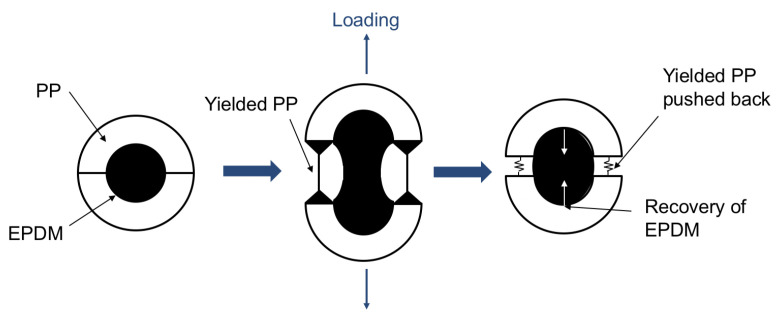
Deformation mechanisms of TPV [57].

The low residual strain obtained after relatively high strains applied indicates that a low level of damage, typically plasticity, takes place during the mechanical cycle in the two specimens. This is in good agreement with Babu et al. [56], who used the TPV deformation model developed by Soliman et al. [57] to explain the behaviour of thermoplastic elastomers vulcanizate. This model explains that the deformation behaviour of TPV is the combination of matrix yielding and buckling because of the recovering tendency of the crosslinked EPDM particles (Figure 9). In our case, the mechanical response was the one of the specimens made of two different TPV. It could be useful to be able to distinguish their single responses during the test, i.e., to access mechanical information at a local scale. This is the reason the next sections focus on the response of each TPV using imaging.

### 3.2. Full Kinematic Field Measurements

**Figure 10:**
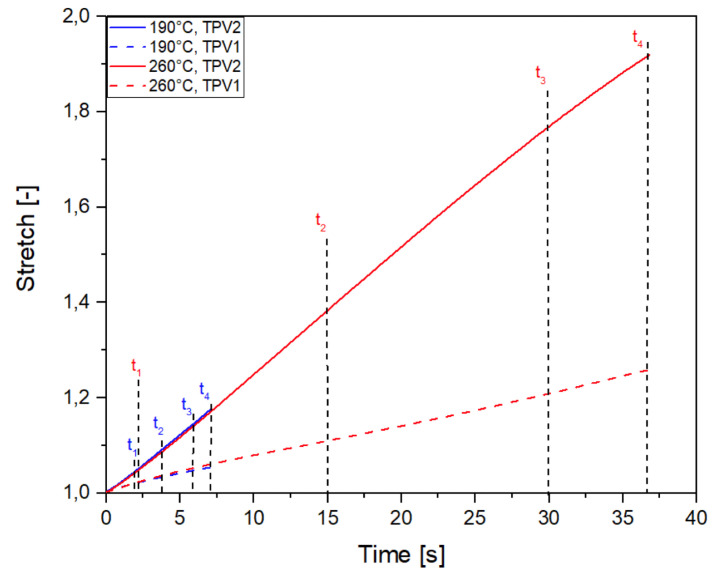
Stretch-time evolution for each TPV overmoulded at $190{\;}^{\circ }C$ and $260{\;}^{\circ }C$, in the case of monotonic tensile test until rupture.

**Figure 11:**
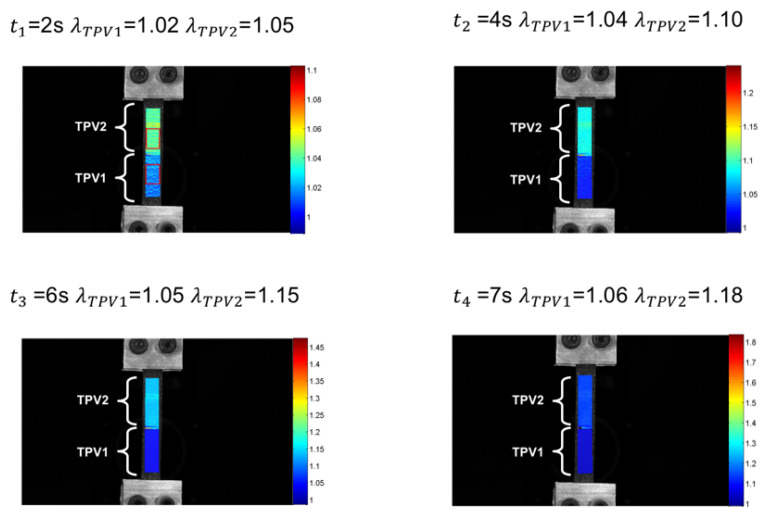
Stretch evolution for each TPV overmoulded at $190{\;}^{\circ }C$, in the case of monotonic tensile test until rupture.

**Figure 12:**
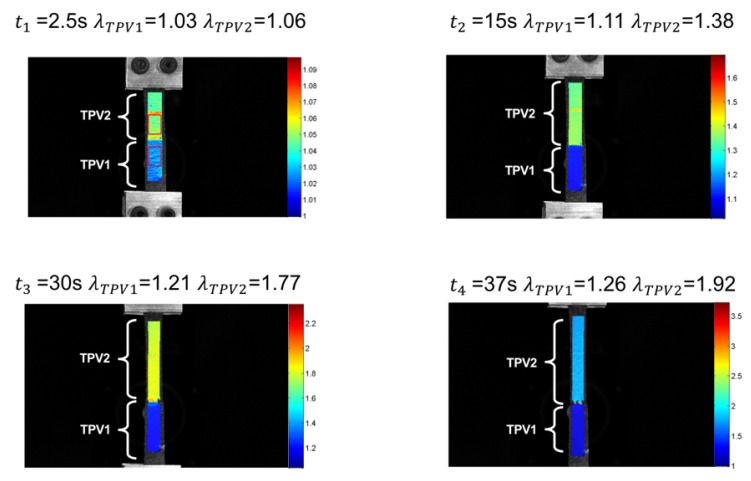
Stretch evolution for each TPV overmoulded at $260{\;}^{\circ }C$, in the case of monotonic tensile test until rupture.

**Figure 13:**
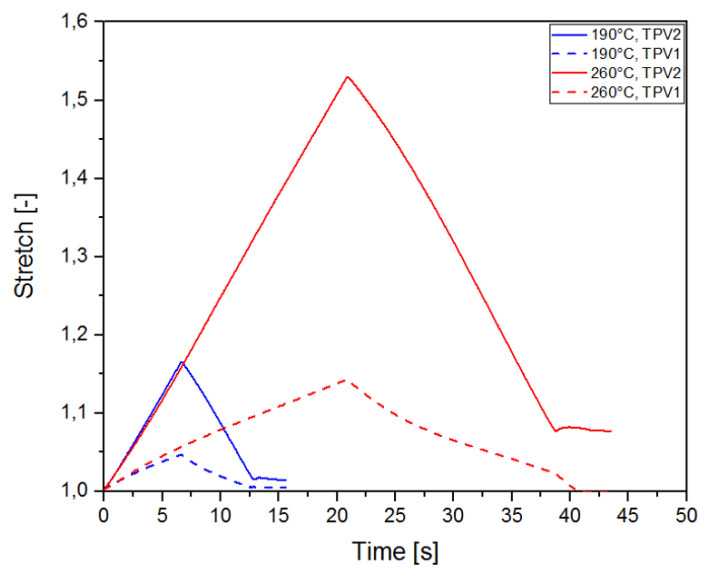
Stretch-time evolution for each TPV overmoulded at $190{\;}^{\circ }C$ and $260{\;}^{\circ }C$, in the case of mechanical cycle.

The DIC technique enables the determination of the kinematic field for the two constitutive TPV. Figure 10 presents the stretch versus time for both injection temperatures and for monotonic tensile test until rupture. For an injection temperature of $190{\;}^{\circ }C$, the stretch levels reached in each TPVs are lower than those observed for an injection temperature of $260{\;}^{\circ }C$. Referring to Section 3.1.2, this behaviour is explained by the temperature dependence of the diffusion mechanisms of PP macromolecules at the interface. For both injection temperatures, it is observed that the elongation reached in TPV2 at the end of the test is higher than that in TPV1. For an injection temperature of $190{\;}^{\circ }C$, $\lambda_{TPV2}$ = 1.18 versus $\lambda_{TPV1}$ = 1.06 (Figure 11), and for an injection temperature of $260{\;}^{\circ }C$, $\lambda_{TPV2}$ = 1.92 against $\lambda_{TPV1}$ = 1.26 (Figure 12). Figure 13 presents the stretch versus time for both injection temperatures and for mechanical cycle tests. From these two figures, it should be noted that TPV2 is much softer than TPV1. Therefore, the whole specimen made with these two TPV is not submitted to a homogeneous loading and consequently, the interface failure cannot be seen theoretically as a crack propagating within a homogeneous medium, meaning that it is complicated to apply the existing models of fracture mechanics to solve interface failure problems [58,59,60].

**Figure 14:**
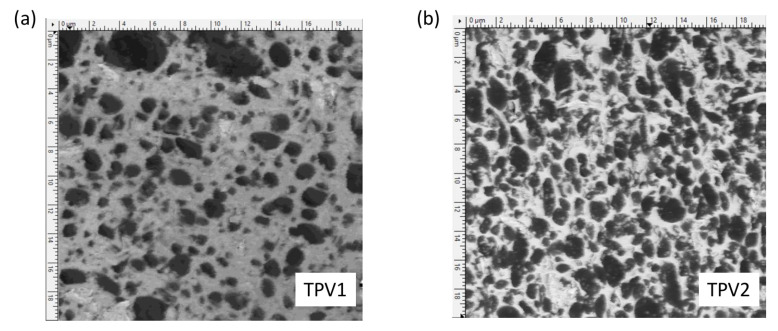
AFM phase image of TPV under study: (**a**) TPV1, injection grade. (**b**) TPV2, extrusion grade. The bright areas correspond to the thermoplastic phase (PP) and the dark areas correspond to the elastomer phase (EPDM). (images size (20 × 20) $\mu m^{2}$).

The elastic properties of the two TPV are very different. This is partly due to their compositions, in particular, their respective proportions of PP and EPDM. Figure 14a,b present an AFM phase image of TPV1 and TPV2, respectively. TPV1 and TPV2 were previously cryo-microtomed from the $190{\;}^{\circ }C$ overmoulded specimens. The phase images can be used to visualize the distribution of the EPDM phase (dark areas) in the PP matrix (bright areas) [61]. TPV1 has larger elastomer nodules and a higher proportion of PP than TPV2. This implies different mechanical responses as shown in Figure 10 and Figure 13. These observations are also in good agreement with the hardness ratio of both TPV presented in Table 1.

### 3.3. Full Temperature Field Measurement

**Figure 15:**
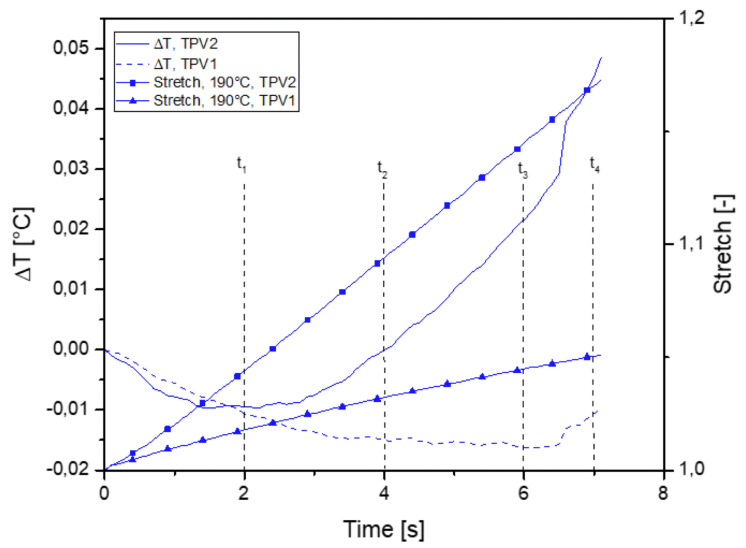
Temperature change with stretch for specimen overmoulded at $190{\;}^{\circ }C$, in the case of monotonic tensile test until rupture.

**Figure 16:**
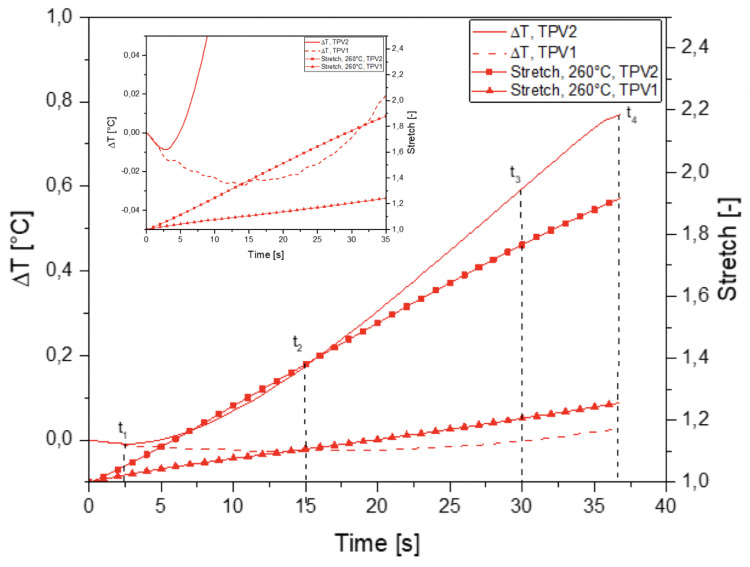
Temperature change with stretch for specimen overmoulded at $260{\;}^{\circ }C$, in the case of monotonic tensile test until rupture.

Figure 15 shows the thermal response measured at the surface of the two TPV in specimen overmoulded at $190{\;}^{\circ }C$ for the tensile test at failure. It is observed that the temperature variation first decreases in the two materials. As the laboratory temperature is stabilised, the only explanation is that the isentropic coupling dominates the thermal response. Furthermore, given the very small temperature variations measured, the intrinsic dissipation caused by the viscosity is very low, even negligible. From a certain strain level, the curve slopes decrease and are inverted in the case of TPV2 for a stretch equal to 1.05. Such a temperature evolution is in a strong analogy with what is observed in elastomers and is generally referred to as the thermoelastic inversion [31,39]. In addition, it is possible that the level of intrinsic dissipation due to viscosity also increases. Figure 16 shows the response obtained with the $260{\;}^{\circ }C$ specimen. Overall, we find the same trends as for the specimen $190{\;}^{\circ }C$ as shown in Figure 16. Indeed, when zooming in on the first part of the curves, i.e., at the lowest strains, the order of magnitude of temperature variation is the same. It should be noted that this increase in temperature can also be induced by a greater viscosity of the materials and therefore a greater intrinsic dissipation. As the interface is more resistant at $260{\;}^{\circ }C$, the level of deformation reached in both materials is greater. This leads to one order of magnitude greater temperature variation in TPV1 between the $190{\;}^{\circ }C$ and $260{\;}^{\circ }C$ specimens. This issue will be addressed in more detail during the analysis of the cyclic tests.

**Figure 17:**
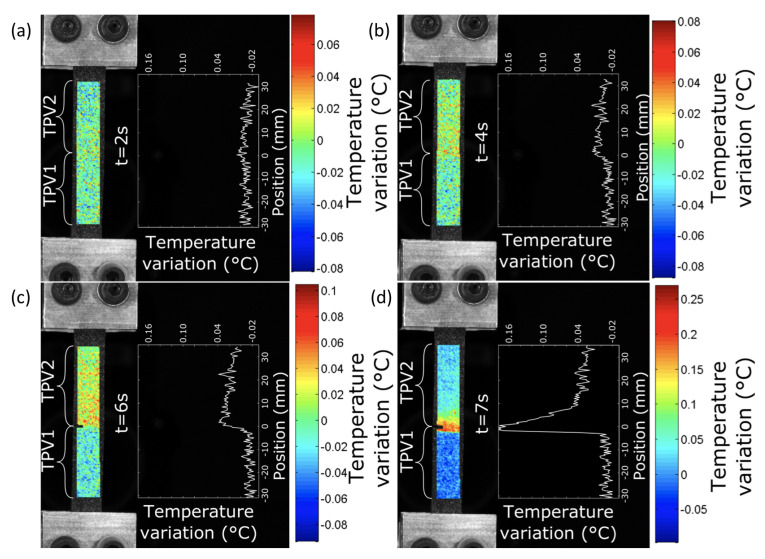
Temperature variation fields and the temperature variation profiles along the specimens overmoulded at $190{\;}^{\circ }C$. (**a**) t${}_{1}$ = 2 s, (**b**) t${}_{2}$ = 4 s, (**c**) t${}_{3}$ = 6 s and (**d**) t${}_{4}$ = 7 s.

**Figure 18:**
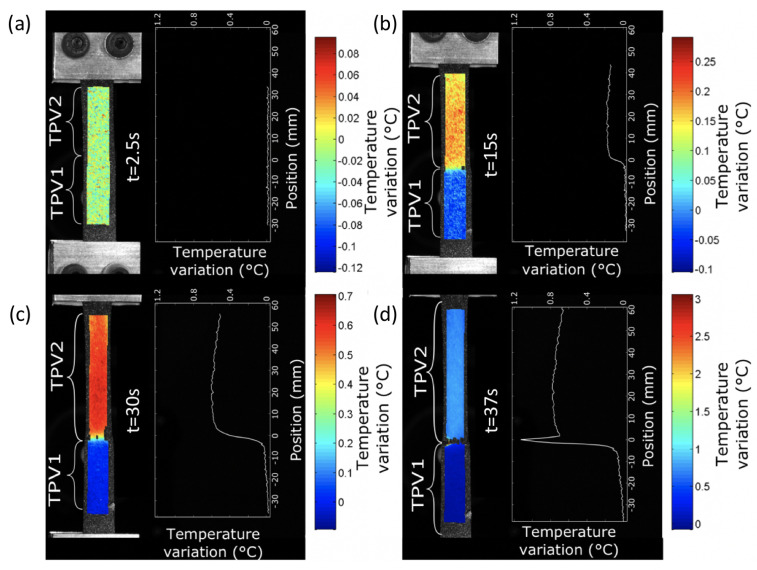
Temperature variation fields and the temperature variation profiles along the specimens overmoulded at $260{\;}^{\circ }C$. (**a**) t${}_{1}$ = 2.5 s, (**b**) t${}_{2}$ = 15 s, (**c**) t${}_{3}$ = 30 s and (**d**) t${}_{4}$ = 37 s.

The analysis of the average temperature variation during the test in each of the two materials far from the interface, i.e., without thermal gradients induced by the stress and strain concentrations of the interface, enabled us to show that the elasticity of the TPVs constituting the specimen is both entropic and isentropic. A competition between the two types of elastic couplings occurs at the low strains. The isentropic coupling is first preponderant, then the entropic coupling takes over. Nevertheless, this analysis does not allow the study of the mechanical behaviour and the effects of the interface in the different materials to be further investigated. This is the reason Figure 17 and Figure 18 show the temperature variation fields and the temperature variation profiles along the specimens at $190{\;}^{\circ }C$ and $260{\;}^{\circ }C$, respectively. Please note that these profiles were determined by averaging the temperature variations across the width. The temperature profiles along the specimen length show a different thermal response for the two TPV as detailed previously. For the smallest and moderate strains (t = 2, 4, 6 and 7s for specimen at $190{\;}^{\circ }C$, and t = 2.5, 15, 30 and 37s for specimens at $260{\;}^{\circ }C$), the thermal response is different from one TPV to another, but it is homogeneous in each TPV. The interfaces do not have a specific thermal signature. Nevertheless, for the highest strains, i.e., close to those at failure, the thermal field exhibits a strong singularity in the interface zone. Indeed, a strong increase in temperature occurs in this zone and can be considered to be a precursor of the interface failure. Such temperature increase is observed in many materials when damage takes place [62]. Please note that the increase in temperature is mainly observed in TPV2. The zone where the temperature is observed is larger (approximately 15mm) in TPV2 in specimen at $260{\;}^{\circ }C$ than in specimen at $190{\;}^{\circ }C$ (approximately 10mm). It should be noted that the temperature rise is $0.16{\;}^{\circ }C$ and $1.2{\;}^{\circ }C$ for the overmoulded samples at $190{\;}^{\circ }C$ and $260{\;}^{\circ }C$, respectively. The fact that an increase in temperature is mainly observed in TPV2 before failure can be interpreted as the effect of an increase of intrinsic dissipation due to internal friction accompanying PP chain disentanglements. Nevertheless, additional experiments must be carried out to further investigate the physical origin of this increase in temperature rise.

**Figure 19:**
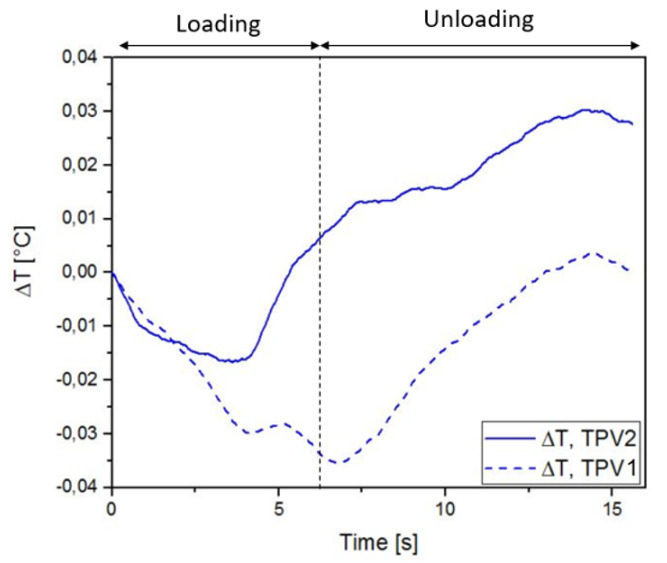
Temperature change with time for specimen overmoulded at $190{\;}^{\circ }C$, in the case of cyclic loading.

**Figure 20:**
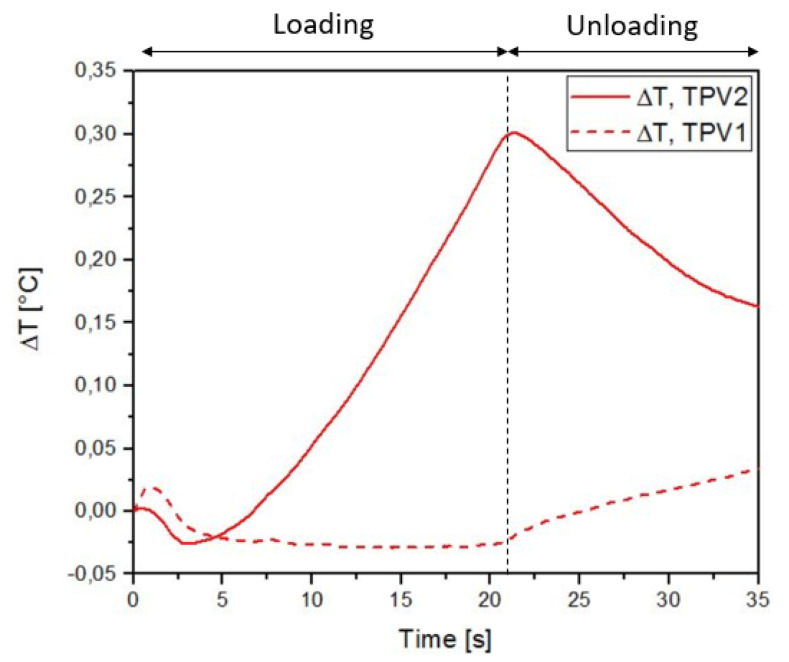
Temperature change with time for specimen overmoulded at $260{\;}^{\circ }C$, in the case of cyclic loading.

Figure 19 and Figure 20 show the thermal response of the overmoulded specimens at $190{\;}^{\circ }C$ and $260{\;}^{\circ }C$ respectively for the mechanical cycle. The thermoelastic inversion for TPV2 is observed for both overmoulding temperatures during the loading phase. For the $190{\;}^{\circ }C$ overmoulded specimens, the TPVs temperature is increasing during the unloading phase reflecting intrinsic dissipations due to viscosity and/or damage. For the $260{\;}^{\circ }C$ overmoulded specimens, the temperature of TPV1 is increasing. Temperature is decreasing for TPV2, meaning that the heat produced due to viscosity decreases.

### 3.4. Prediction of Adhesion

**Figure 21:**
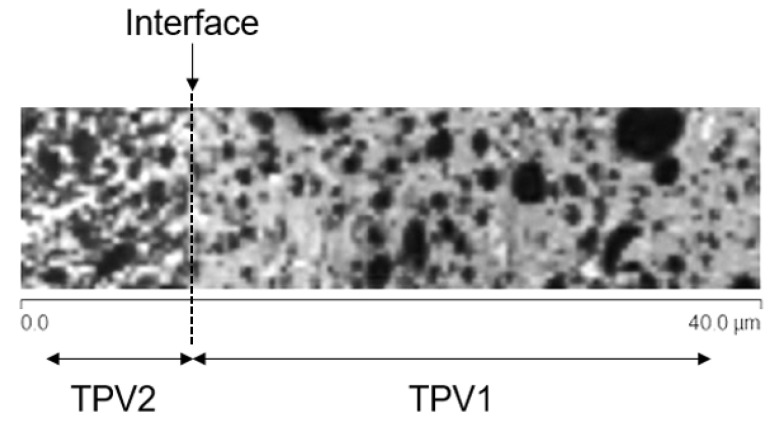
AFM phase image: TPV1-TPV2 interface observation by AFM.

The failure observed at a lower strength level in the case of the $190{\;}^{\circ }C$ specimens in Figure 4 may be explained regarding the overmoulding process. Assuming diffusion of the molten TPV1 into TPV2 insert, the strength of the overmoulded specimen is increasing with injection temperature because of the decreasing value of reptation time with temperature (Equation (2)) [63]. In the following considerations, we assume an instantaneous intimate contact. We will focus on the calculation of the degree of healing based on a rheological determination of relaxation times of PP representative of the matrix of both TPVs. Indeed, PP can be considered to be the main material in the composition of the TPV contributing to adhesion, as observed by AFM as presented in Figure 21. TPV2 is located on the left side and TPV1 on the right one. The interface between the two overmoulded TPVs is represented by dotted lines and is made of PP. The mechanical strength of the assembly of the two overmoulded TPVs is thus linked to the entanglements formed by the PP chains that will renew their configuration and cross the interface.

#### 3.4.1. Rheological Characterization

The mastercurve was determined at $200{\;}^{\circ }C$ from an Arrhenius plot of the shift factors a${}_{T}$ versus the inverse of the temperature. The activation energies were E${}_{a,PP1,2}$ = 41.94kJ/mol and E${}_{a,PP1}$ = 39.76kJ/mol for PP1,2 and PP1, respectively. and are in good agreement with the literature [64,65]. The mastercurve was fitted with a *N* = 5 mode Maxwell model (Equations (5) and (6)).

(5) $$G{}^{\prime }\left(\omega \right)=\sum_{i=1}^{N}G_{i}\frac{{\left(\omega \tau_{i}\right)}^{2}}{1+{\left(\omega \tau_{i}\right)}^{2}}$$

(6) $$G{}^{\prime \prime }\left(\omega \right)=\sum_{i=1}^{N}G_{i}\frac{\omega \tau_{i}}{1+{\left(\omega \tau_{i}\right)}^{2}}$$

The relaxation moduli G${}_{i}$ and the associated relaxation time $\tau_{i}$ were calculated at $200{\;}^{\circ }C$. The average relaxation time given by $\tau_{w}=\frac{\sum G_{i}\tau_{i}^{2}}{\sum G_{i}\tau_{i}}$. At $200{\;}^{\circ }C$, $\tau_{w}$ = 1.37s for PP1 and $\tau_{w}$ = 0.80s for PP1,2. The values obtained were compared to the Cole-Cole plot. From the plot of imaginary $\eta {}^{\prime \prime }$ versus real $\eta {}^{\prime }$ components of the complex viscosity one can determine a weight-average relaxation time from the frequency at the maximum of η${}^{\prime \prime }$. The average relaxation time $\tau_{w}$ obtained by this technique for PP1,2 is approximatively 0.63s and 1.23s for PP1. The differences in the calculated values can be explained by the polydispersity of both polypropylenes, i.e., they have a distribution of relaxation times reflecting the whole chains dynamics. The relaxation times obtained are then extrapolated with an Arrhenius relation using the activation energies.

#### 3.4.2. Thermophysical Properties

**Figure 22:**
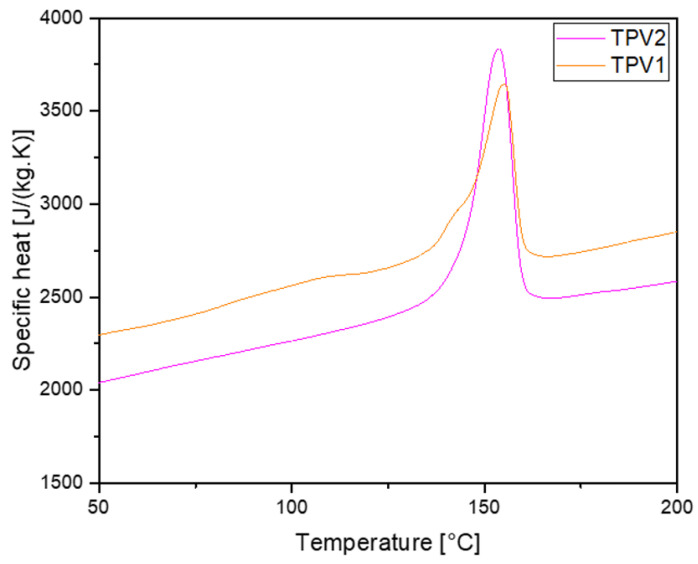
Apparent specific heat versus temperature for our two grades of TPV.

The calculation of the relaxation times needs to be coupled with the simulation of the cooling of the overmoulded part to predict the degree of healing. The modelling of heat transfer during cooling in semicrystalline polymer requires the knowledge of thermophysical properties as a function of temperature. Figure 22 presents the apparent heat capacity of the two TPV versus temperature. Between $125{\;}^{\circ }C$ and $175{\;}^{\circ }C$, the two peaks correspond to the melting of the PP matrices.

Thanks to these measurements, $C_{p}$ has been evaluated in J/(kg.K) for the two TPV as a function of temperature *T* in ${\;}^{\circ }C$. The results are presented in Table 5.

**Figure 23:**
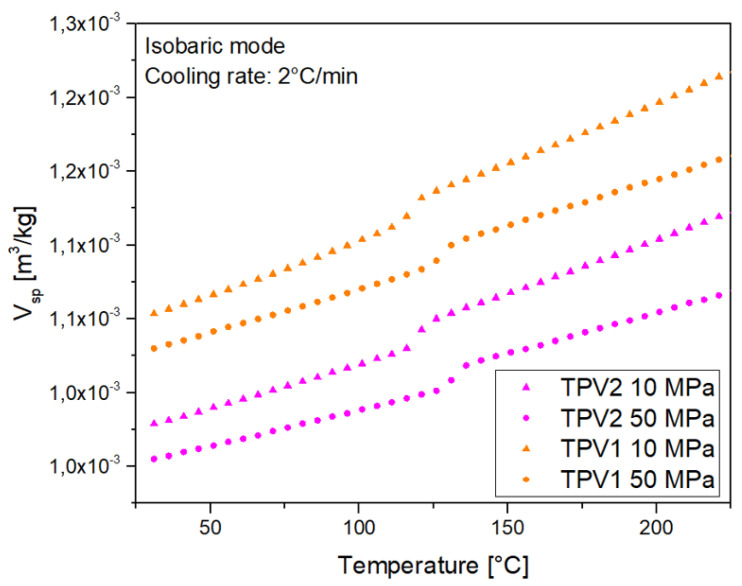
PVT diagram for TPV1 and TPV2 at two pressure levels.

The PVT diagram of TPV1 and TPV2 is presented in Figure 23. TPV1 has a lower density than TPV2 for both isobars. The data are fitted following a linear regression in the molten and in the solid states. The results are presented in Table 6.

**Figure 24:**
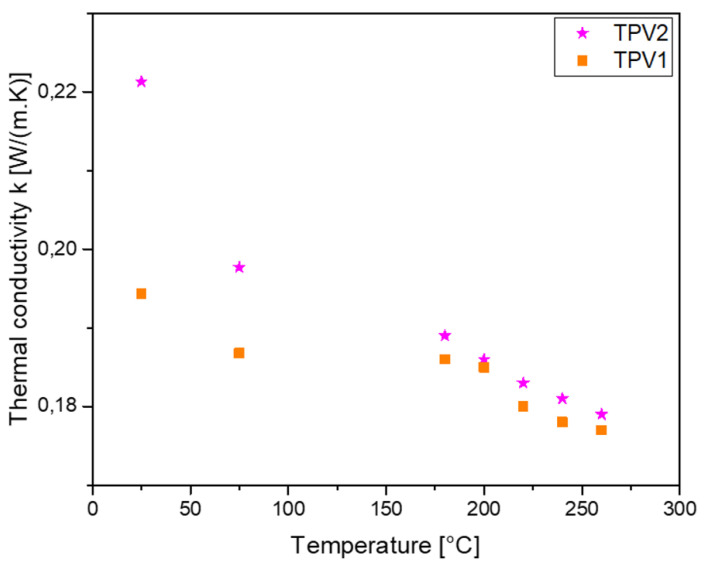
Thermal conductivity versus temperature for TPV1 and TPV2.

Figure 24 presents the results obtained for the two TPV. The data are fitted following a linear regression, as presented in Table 7.

#### 3.4.3. Prediction of the Interface Temperature during Overmoulding

**Figure 25:**
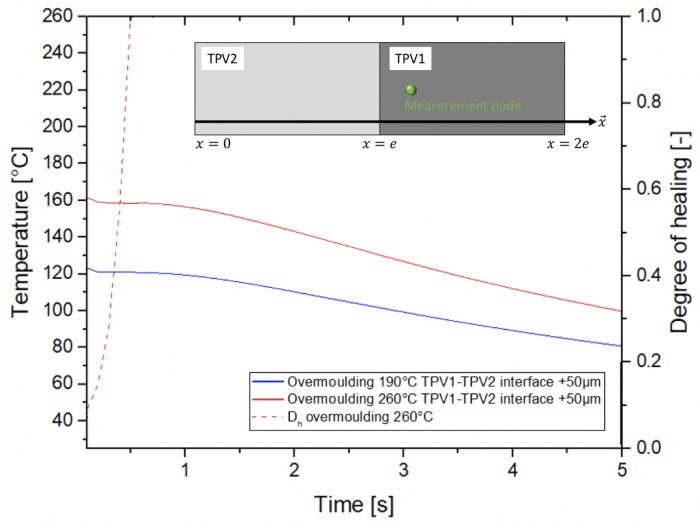
Temperature evolution for a node located at x=e+ 50μm from interface in TPV1.

The relaxation time thus obtained make it possible to calculate the degree of healing as a function of the temperature field during the cooling of the overmoulded part. In the following, we assume 1D heat transfer through the overmoulded part. TPV2 is located between x=0 and x=e and TPV1 between x=e and x=2e (Figure 25). The cooling of the TPV1 was modelled by the 1D heat transfer equation (Equation (7)) through the part (e<x<2e). At the initial time, the temperature field was assumed to be uniform in TPV1 and equal to the injection temperature T${}_{inj}$ (Equation (11)). The TPV1 crystallization rate was modelled by the differential form of Nakamura (Equation (8)) [66,67], and a heat source term was added in the heat transfer equation (Equation (7)). A non-linear solving system was used to take into account the thermodependency of thermal properties and crystallization.

(7) $$\begin{array}{c} \frac{\partial }{\partial x}\left(k_{TPV1}\frac{\partial T_{TPV1}}{\partial x}\right)+\rho_{TPV1}\Delta H\frac{\partial \alpha }{\partial t}=\rho_{TPV1}C_{p,TPV1}\frac{\partial T_{TPV1}}{\partial t}\;for\;e<x<2e\\ and\;\forall t>0\;\; \end{array}$$

where ΔH is the crystallization enthalpy.

(8) $$\frac{\partial \alpha }{\partial t}=n\cdot K_{Nak}\cdot \left(1-\alpha \right)\cdot {\left(-ln\left(1-\alpha \right)\right)}^{\frac{\left(n-1\right)}{n}}$$

(9) $$+k_{TPV1}\frac{\partial T_{TPV1}\left(2e,t\right)}{\partial x}=\phi_{mould}\left(t\right)\;at\;x=2e$$

(10) $$-k_{TPV1}\frac{\partial T_{TPV1}\left(e+,t\right)}{\partial x}=\phi_{1}\left(t\right)\;at\;x=e+$$

(11) $$T_{TPV1}\left(x,t=0\right)=T_{inj}\;for\;e\leq x\leq 2e$$

The cooling of the TPV2 was modelled by the heat transfer (Equation (12)) through the part (0<x<e). At the initial time, the temperature field was assumed to be uniform and equal to the mould temperature T${}_{ex}$ (Equation (15)). It is assumed that the TPV2 does not undergo a phase change.

(12) $$\frac{\partial }{\partial x}\left(k_{TPV2}\frac{\partial T_{TPV2}}{\partial x}\right)=\rho_{TPV2}C_{p,TPV2}\frac{\partial T_{TPV2}}{\partial t}\;for\;0<x<e$$

(13) $$-k_{TPV2}\frac{\partial T_{TPV2}\left(0,t\right)}{\partial x}=\phi_{mould}\left(t\right)\;at\;x=0$$

(14) $$-k_{TPV2}\frac{\partial T_{TPV2}\left(e-,t\right)}{\partial x}=\phi_{1}\left(t\right)\;at\;x=e-$$

(15) $$T_{TPV2}\left(x,t=0\right)=T_{ex}\;for\;0\leq x\leq e$$

The continuity of heat flux densities at the interface is expressed as (Equation (16)):

(16) $$-k_{TPV1}\frac{\partial T_{TPV1}\left(e+,t\right)}{\partial x}=-k_{TPV2}\frac{\partial T_{TPV2}\left(e-,t\right)}{\partial x}=\frac{T_{TPV2}\left(e-,t\right)-T_{TPV1}\left(e+,t\right)}{TCR}$$

We considered for the simulation a constant thermal contact resistance (TCR) value at the TPVs interface ($4\times 10^{-3}\;K\;m^{2}/W$). This value was taken as equal to the TCR determined by Somé et al. [68] at PP/mould interface since TCR at polymer interfaces during the overmoulding process appears to be non-existent in the literature to the author’s knowledge. Before overmoulding, the surface roughness of TPV2 was measured (Mitutoyo SJ-301) at R${}_{a}$ = 0.43μm. The cooling profiles of TPV1 are then calculated at x=e+50μm from the interface, i.e., outside the thermally disturbed zone.

Figure 25 presents the cooling profiles calculated at a position located at 50μm from interface in TPV1 for injection temperatures of $190{\;}^{\circ }C$ and $260{\;}^{\circ }C$. The degree of healing was only calculated for the case of a $260{\;}^{\circ }C$ overmoulding since in this configuration the TPV1 and TPV2 are melted at the interface. The average of the Maxwell model relaxation times of PP1 and PP1,2 was considered for the calculations. In the case of an overmoulding at $190{\;}^{\circ }C$, only the TPV1 is melted at the interface. The degree of healing cannot be calculated since TPV2 remains in the solid state. Referring to the work of Nguyen et al. [69], the adhesion of TPV1 to TPV2 is carried out by Rouse-type chain movements of PP1,2 in the amorphous regions of PP1 which is in the semicrystalline state. The adhesion levels observed in this case are lower than those observed for partial TPV1/TPV2 interface remelting, i.e., in the case of a $260{\;}^{\circ }C$ injection melt temperature, where the degree of healing reaches a maximum value for t ≈0.5s.

## 4. Conclusions

This paper presents the first thermomechanical study of an assembly of two soft TPV obtained by overmoulding process and their interface. The rheological and thermophysical properties of the two TPV have first been fully characterized. Then, the mechanical tests carried out with the assemblies have highlighted that under monotonous tensile loading, the failure occurs at the interface. The DIC measurements have shown that the TPVs have different stiffnesses, which leads to a significant change in the interface position along the specimens during stretching. Furthermore, these measurements have shown that a significant necking is observed in the softer material and that the zone of influence of the interface in terms of stretch gradient is very different in size from one material to the other. Therefore, the interface resistance can be addressed neither as a crack propagating in a homogeneous medium nor a crack propagating at the interface between a soft and a rigid body. These questions undoubtedly on how to apply fracture mechanics on overmoulded soft–soft materials with a significant difference in stiffness. In addition, thermal investigations have shown that the elasticity of the two TPV is due to both entropic and non-entropic effects, the former being the most significant at large strains. Moreover, for high strain levels, the interface exhibits a singular behaviour characterized by a significant rise in temperature. The thermally influenced zone is larger for specimens moulded at higher temperatures. The heat production is even more important as the resistance of the interface is important. Analysis of average temperature profiles close to the interface reveals a strong heat production when the rupture occurs. In addition, the influence of the overmoulding temperature has been studied. It was shown that the increase in injection temperature induced an increase in the strength of the overmoulded part. Assuming a diffusion of the polypropylene macromolecules present at the interfaces, an acceleration of the adhesion kinetics was shown with the increase of the injection temperature.

This information could be used in future work to implement predictive models of the thermomechanical behaviour of soft–soft overmoulded junctions.

## Figures and Tables

**Table 1:** Main properties of the studied materials.

Properties	TPV1	TPV2
Hardness [Shore A]	81	72
Elongation at break, $23{\;}^{\circ }C$ [%]	610	450
Young modulus, $23{\;}^{\circ }C$ [MPa]	25.5	19.6
Tensile stress at 100%, $23{\;}^{\circ }C$ [MPa]	4.1	3.0

**Table 2:** Molar mass, polydispersity index and activation energy of PPs under study.

Material	$M_{n}$ [g/mol]	$M_{w}$ [g/mol]	$I_{p}$ [−]
PP1,2	80,528	167,612	1.8
PP1	96,800	197,600	2.0

**Table 3:** DIC Hardware parameters.

DIC Hardware Parameters	Detail
Camera	IDS UI-3160CP Rev. 2
Image Resolution	( 1920 × 1200 ) px
Lens	55 mm C-mount partially telecentric.
	Constant magnification over a range
	of working distances ±12.5 mm
	of object movement before 1% error
	image scale occurs
Field-of-View	(186.8 × 116.8) mm${}^{2}$
Image Scale	10px/mm
Stand-off Distance	1500mm
Image Acquisition Rate	10Hz
Patterning Technique	White spray on black specimen
Pattern Feature	
Size (approximation)	6 px

**Table 4:** DIC Software parameters.

DIC Analysis Parameters	Detail
DIC Software	7D©
Image Filtering	None
Subset Size	8 px/0.78mm
Step Size	4 px/0.39mm
Subset Shape Function	Affine
Matching Criterion	Zero-Mean Normalized Cross Correlation
Interpolant	Bi-cubic
Strain Window	5 data points
Virtual Strain Gauge Size	24 px/2.34mm
Strain Formulation	Principal Maximal
Post-Filtering of Strains	None
Displacement Noise-Floor	0.011 px/10.7μm
Strain Noise-Floor	0.0023

**Table 5:** Apparent specific heat of TPVs under study.

	TPV1		TPV2	
	$C_{p}$ [*J*/(kg.K)]	R${}^{2}$	$C_{p}$ [*J*/(kg.K)]	R${}^{2}$
Solid state	5.22T + 2026	0.99	4.53T + 1815	0.99
Amorphous state	4.19T + 2011	0.99	2.79T + 2023	0.99

**Table 6:** Specific volume of TPVs under study.

	TPV1		TPV2	
	$V_{\mathrm{sp}}$ [m${}^{3}$/kg]	R${}^{2}$	$V_{\mathrm{sp}}$ [m${}^{3}$/kg]	R${}^{2}$
Solid state	$7.25\times 10^{-7}$ T + $1.08\times 10^{-3}$	0.99	$6.01\times 10^{-7}$ T + 1.01.10${}^{-3}$	0.99
Amorphous state	$8.20\times 10^{-7}$ T + $1.07\times 10^{-3}$	0.99	$7.27\times 10^{-7}$ T + $1.01\times 10^{-3}$	0.99

**Table 7:** Thermal conductivity of TPVs under study.

TPV1		TPV2	
k **[W/(m.K)]**	**R** ${}^{2}$	k **[W/(m.K)]**	**R** ${}^{2}$
$-7.3\times 10^{-5}$ T + 0.197	0.90	$-1.8\times 10^{-4}$ T + 0.225	0.99

## Data Availability

Data available on request due to restrictions eg privacy or ethical.

## Abbreviations

The following abbreviations are used in this manuscript:

TPV	Vulcanizate Thermoplastic Elastomers
EPDM	Ethylene-Propylene-Diene-Monomer
TPE	Thermoplastic Elastomers
EWIF	Essential Work of Interfacial Fracture
TPU	Thermoplastic Polyurethane
MABS	Methylmethacrylate-Butadiene-Styrene Copolymer
AFM	Atomic Force Microscope
FDM	Fused Deposition Modelling
CCD	Charge-Coupled Device
DIC	Digital Image Correlation
ZOI	Zone Of Interest
NETD	Noise Equivalent Temperature Difference
PS	Pure Shear
EQT	Equibiaxial Tension
UT	Uniaxial Tension
IR	Infrared
NUC	Non-Uniformity Correction
